# Supplementary material for: Dopamine signaling drives skin invasion by human-infective nematodes
Source: Nat Commun. 2025 Aug 13;16:7246. doi: 10.1038/s41467-025-62517-z (PMC12350745; doi:10.1038/s41467-025-62517-z)
Supplement: Supplementary file 1 — Supplementary Information [file 41467_2025_62517_MOESM1_ESM.pdf]

SUPPLEMENTARY INFORMATION:

Dopamine signaling drives skin invasion by human-infective nematodes

Ruhi Patel[1], Gloria Bartolo[1,2], Michelle L. Castelletto[1], Aracely Garcia Romero[1], Astra S. Bryant[3], George W. Agak[4,5], and Elissa A. Hallem[1,5,*]

[1] Department of Microbiology, Immunology, and Molecular Genetics, University of California, Los Angeles, Los Angeles, CA 90095, USA

[2] Molecular Biology Interdepartmental PhD Program, University of California, Los Angeles, Los Angeles, CA 90095, USA

[3] Department of Neurobiology and Biophysics, University of Washington, Seattle, WA 98195, USA

[4] Division of Dermatology, Department of Medicine, David Geffen School of Medicine at University of California, Los Angeles, Los Angeles, CA 90095, USA

[5] Molecular Biology Institute, University of California, Los Angeles, Los Angeles, CA 90095, USA

*Corresponding author: ehallem@ucla.edu

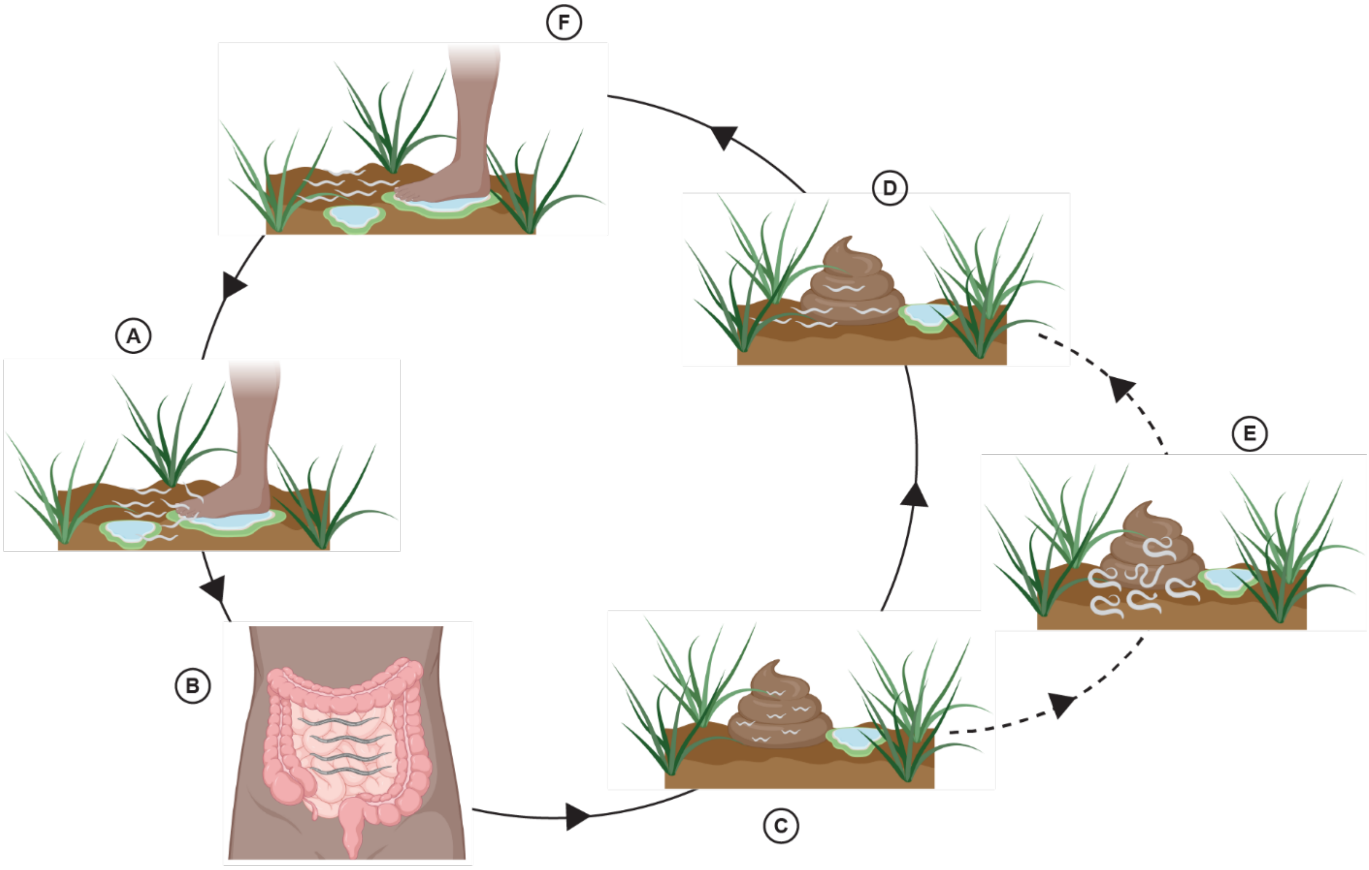

Fig. S1. The life cycle of *S. stercoralis.* The life cycle of *S. stercoralis* is composed of both intra-host and extra-host life stages[1]. The intra-host portion of the life cycle begins when infective third-stage larvae (iL3s) penetrate the skin of a host and enter the body (A); the natural hosts of *S. stercoralis* include humans (as depicted in the figure), some non-human primates, and dogs[1-3]. Development is paused in iL3s and resumes upon host entry[4]. Following skin penetration, larvae travel through the body and reach the host duodenal mucosa, where they live and reproduce as parasitic adults[5] (B). Parasitic adults lay eggs in the duodenal mucosa[5], which hatch into post-parasitic, first-stage (L1) larvae. A subset of the post-parasitic L1s develop into autoinfective third-stage larvae (aL3s), which can reinfect the host and perpetuate the *S. stercoralis* infection (not shown). Alternatively, post-parasitic L1s are released from the host, into the surrounding environment, in feces (C). Outside of the host, post-parasitic L1s are fated to one of two developmental routes: they either develop directly into iL3s (D) or they develop into free-living females and males (E). All the progeny of free-living females and males become iL3s. The iL3s actively seek out new hosts using cues such as heat and host-emitted odorants[6] (F). The life cycle of *S. ratti* is very similar to *S. stercoralis*, except that *S. ratti* infects rats and all the progeny of parasitic adults exit the host as eggs (thus, *S. ratti* does not have an autoinfective cycle). Created in BioRender. Mushtaqh Ali, R. (2025) https://BioRender.com/hrxuc96

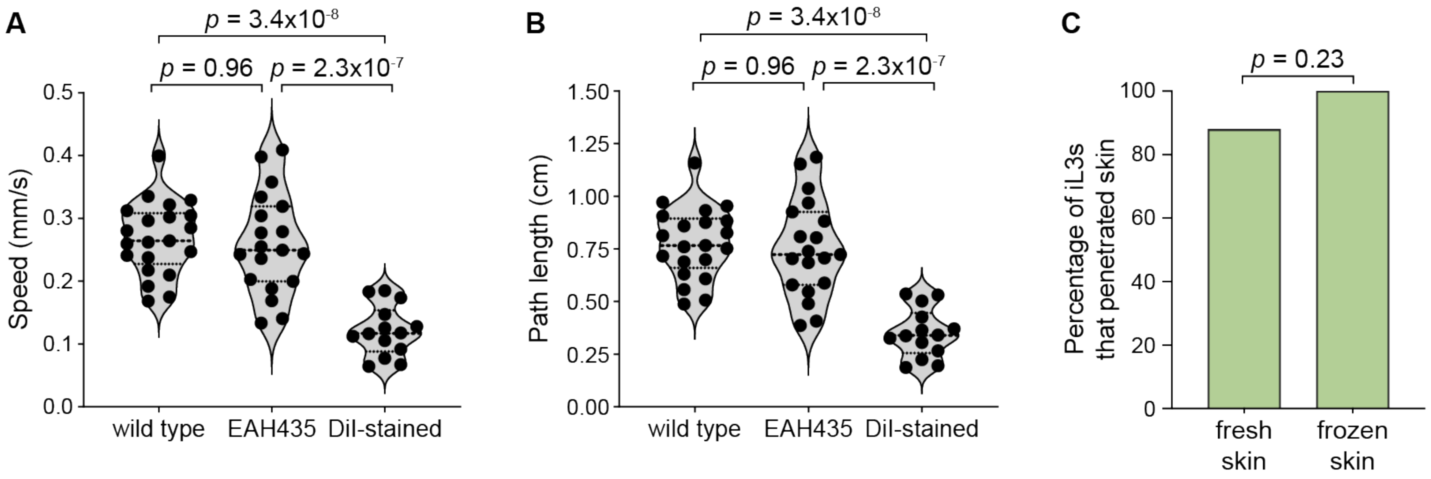

Fig. S2. Controls for *ex vivo* skin-penetration assays. A. Wild-type and EAH435 *bruIs4*[*Sst-act-2p::strmScarlet-I::Sst-era-1* 3′ UTR][7] transgenic *S. stercoralis* iL3s moved at roughly the same speed on an agar surface, while DiI-stained *S. stercoralis* iL3s moved more slowly. n = 21 wild-type, 19 EAH435, and 14 DiI-stained iL3s. **B.** Wild-type and EAH435 transgenic *S. stercoralis* iL3s moved a similar distance on an agar surface, while DiI-stained iL3s moved a shorter distance. n = 21 wild-type, 19 EAH435, and 14 DiI-stained iL3s. However, both DiI-stained iL3s and EAH435 transgenic iL3s were equally able to penetrate skin (Fig. 2D, Fig. 3B) and spent similar amounts of time on the surface of rat skin pushing and puncturing the skin (Fig. 2B, Fig. 3C), indicating that their skin-penetration behavior is similar. For A-B, dots depict individual worms, dashed lines indicate medians, and dotted lines indicate interquartile ranges. Behavioral parameters plotted in A-B were obtained from 3 independent replicates. One-way ANOVA with Sidak’s post-test was used for A-B. **C.** *S. stercoralis* iL3s are equally able to penetrate fresh and frozen rat skin. Bar plots show the percentage of *S. stercoralis* EAH435 transgenic iL3s that penetrated fresh or frozen rat skin. Fresh rat skin was obtained from a rat within 2 h of euthanasia and was not frozen prior to use in skin-penetration assays. In contrast, frozen rat skin had been frozen at least once and up to two times before use in skin-penetration assays. n = 25 iL3s on fresh skin and 24 iL3s on frozen skin. Two-tailed Fisher’s exact test was used for statistical testing. Skin from four distinct rats was tested for the fresh skin condition and skin from three distinct rats was tested for the frozen skin condition. There are no error bars because the graph shows the percentage of iL3s on each skin type that completed penetration out of the total tested. Source data are provided in the source data file.

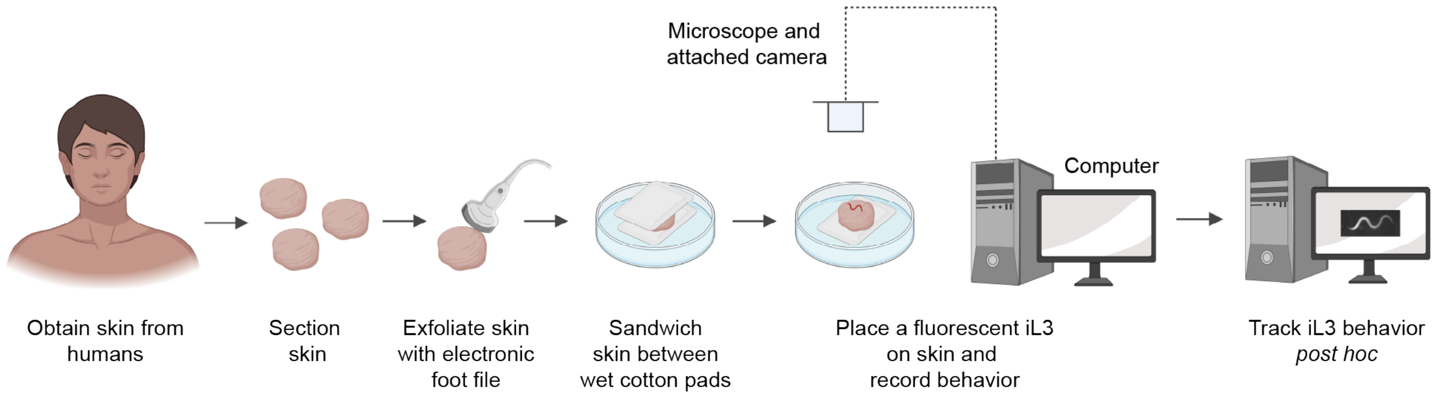

Fig. S3. An *ex-vivo* assay for studying skin-penetration behaviors on human skin. Skin samples are obtained either from cadaver donors (forearm skin) or from patients that underwent surgery (breast or abdominal skin) and then sectioned. Humans often come in contact with *S. stercoralis* when walking barefoot through contaminated soil[8]. The iL3s then penetrate, often through skin on the top surface of the foot or the toes[9], and enter the body. To mimic the relative thinness of the skin from the top surface of the foot and toes[10,11], as well as any micro-abrasions caused by walking barefoot in soil, the skin is exfoliated for 5-15 s with an electronic foot file. The skin is then sandwiched between cotton pads that were pre-moistened with 1X PBS to maintain moisture. Fluorescent iL3s are placed on the skin surface and time-lapse images of behavior are acquired for 10 min thereafter or until penetration is complete. Time-lapse images are recorded using a fluorescence microscope and camera, and videos are analyzed *post hoc*. Created in BioRender. Mushtaqh Ali, R. (2025) https://BioRender.com/zwr70i9

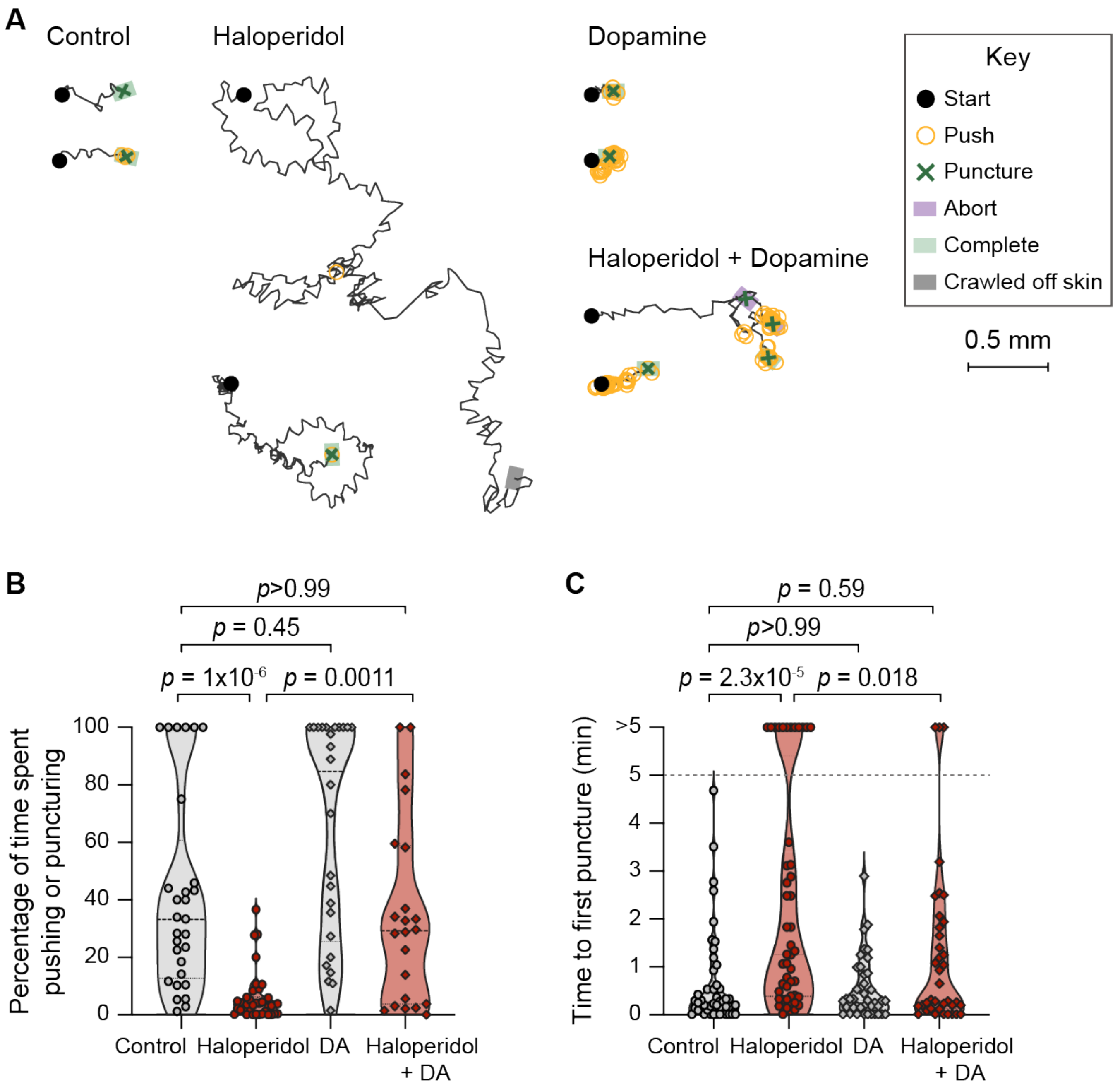

Fig. S4. Pharmacological manipulation of dopamine signaling blocks skin penetration in *S. ratti*. A. Haloperidol inhibits skin penetration and dopamine (DA) rescues this phenotype in *S. ratti* iL3s. Tracks show two representative worms from each group; the haloperidol-treated group shows one representative worm that completed penetration and one that neither punctured nor completed penetration. The haloperidol-treated worm that did not complete penetration crawled off the edge of the skin, as depicted by a gray box. Key shows the behavioral motifs that were tracked. **B.** Haloperidol reduces the percentage of time that *S. ratti* iL3s spend pushing and puncturing the skin, and this effect is rescued by addition of exogenous DA. Violin plot depicts the percentage of time iL3s spent engaging in pushes or punctures. n = 29 control, 36 haloperidol-treated, 26 dopamine-treated, and 22 haloperidol+dopamine-treated iL3s. **C.** Haloperidol delays the time to first puncture and DA rescues this behavioral phenotype. Violin plot depicts the time taken by worms from each treatment group to first puncture the skin. n = 46 control, 48 haloperidol-treated, 41 dopamine-treated, and 38 haloperidol+dopamine-treated iL3s. The dotted line at y = 5 indicates the time at which the assay ended; the dots above this line indicate worms that failed to puncture the skin. For B-C, dots depict individual worms, dashed lines indicate medians, and dotted lines indicate interquartile ranges. Behavioral parameters plotted in B were obtained from 4 independent replicates and those in C were obtained from 5 independent replicates. Kruskal-Wallis test with Dunn's post-test was used for B-C. DiI-stained iL3s and rat skin were used for these experiments. Source data are provided in the Source Data file.

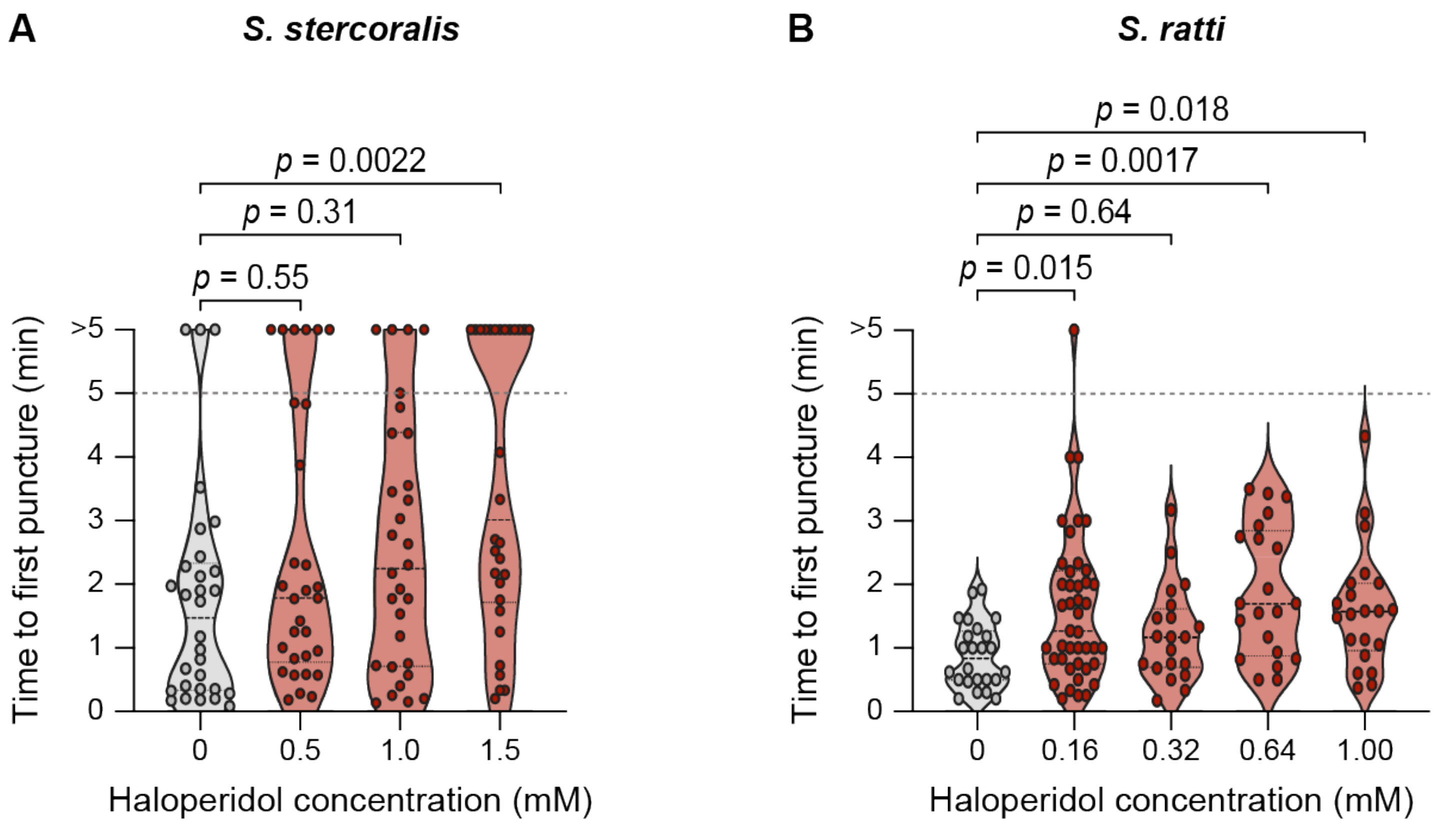

Fig. S5. Effects of different concentrations of haloperidol on *S. stercoralis* and *S. ratti* skin-penetration behavior. A. The time to first puncture was significantly delayed among *S. stercoralis* iL3s treated with 1.5 mM haloperidol, but not at lower concentrations. Violin plot depicts the time taken to first puncture the skin. Vehicle-only control worms are depicted in gray and haloperidol-treated worms are depicted in red. The vehicle-only condition contained 3.8% DMSO, which is the same concentration of DMSO that is present in all of the haloperidol treatment groups. n = 30 iL3s per condition. **B.** The time to first puncture was delayed among *S. ratti* iL3s treated with concentrations of haloperidol as low as 0.16 mM. Violin plot depicts the time taken to first puncture the skin. Vehicle-only control worms are depicted in gray and haloperidol-treated worms are depicted in red. The vehicle-only condition contained 5.0% DMSO, which is the same concentration of DMSO that is present in the 1 mM haloperidol treatment group; all other treatment groups have lower concentrations of DMSO. n = 24 control, 43 0.16-mM-treated, 20 0.32-mM-treated, 21 0.64-mM-treated, and 21 1-mM-treated iL3s. For A-B, dots depict individual worms, dashed lines indicate medians, and dotted lines indicate interquartile ranges. Behavioral parameters plotted in A were obtained from 3 independent replicates and those in B were obtained from 2-3 independent replicates. Kruskal-Wallis test with Dunn's post-test was used for A-B. DiI-stained iL3s and rat skin were used in these experiments. Source data are provided in the Source Data file.

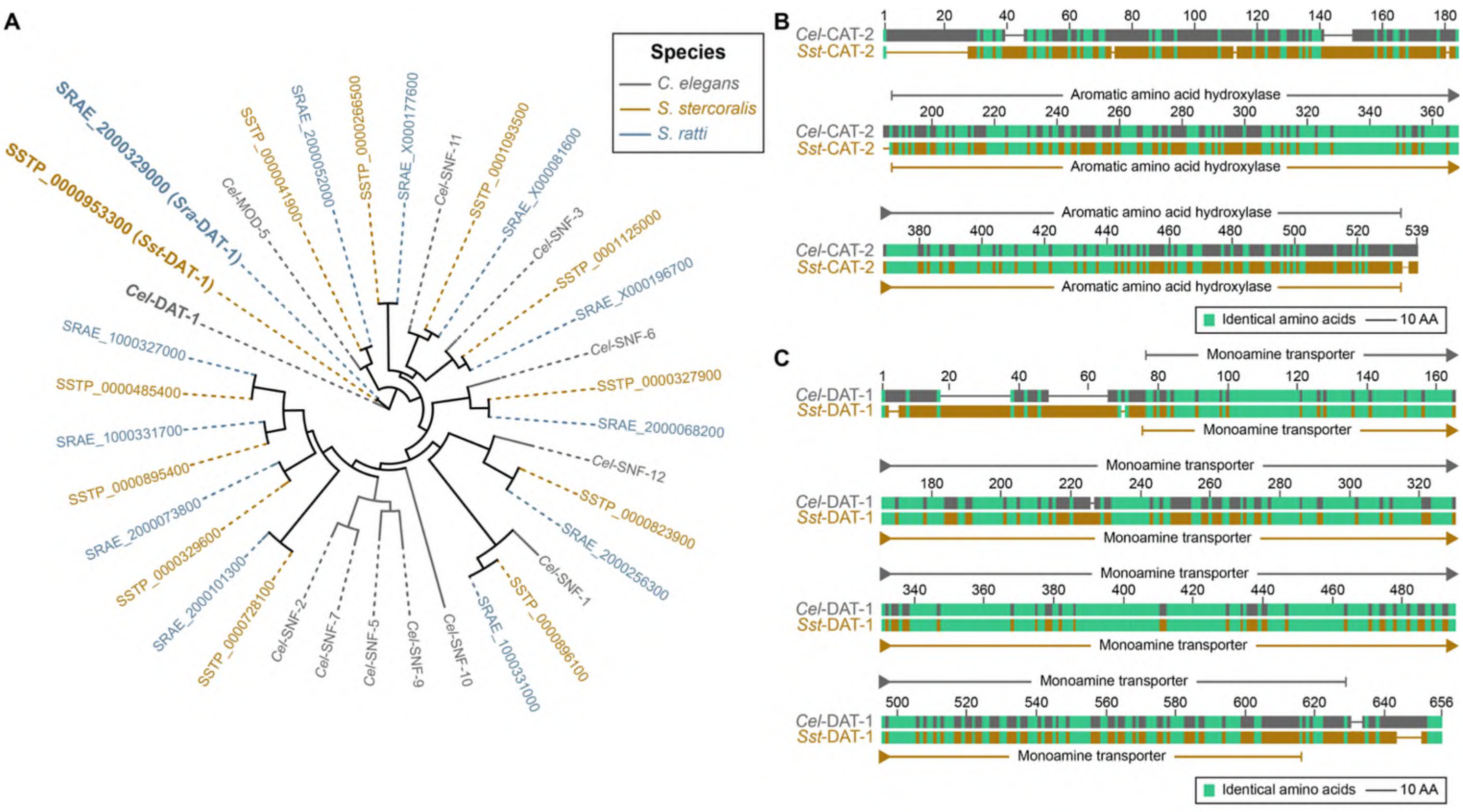

Fig. S6. The *S. stercoralis* homologs of *Cel*-DAT-1 and *Cel*-CAT-2. A. Phylogenetic analysis shows the closest homologs of *C. elegans* DAT-1 (gray), *S. stercoralis* (brown), and *S. ratti* (blue). The tree has all known members of the sodium neurotransmitter symporter family (SNF) of proteins in *C. elegans*, of which DAT-1 is a member, and predicted homologs in *S. stercoralis* and *S. ratti*. **B.** Schematic representation of the alignment of the amino acid sequences of the *C. elegans* CAT-2 protein (isoform A, top) and the *S. stercoralis* CAT-2 protein (bottom). The aromatic amino acid hydroxylase domains, which are the catalytic domains of this family of enzymes[12], of the two proteins are 56% identical. **C.** Schematic representation of the alignment of the amino acid sequences of the *C. elegans* DAT-1 protein (top) and the *S. stercoralis* DAT-1 protein (bottom). The monoamine transporter domains are ~69% identical. In **B**-**C**, identical amino acids are shown in green, and the domains were identified by searches in the Conserved Domain Database[13]. Drawings are to scale and the scale bar = 10 amino acids (AA).

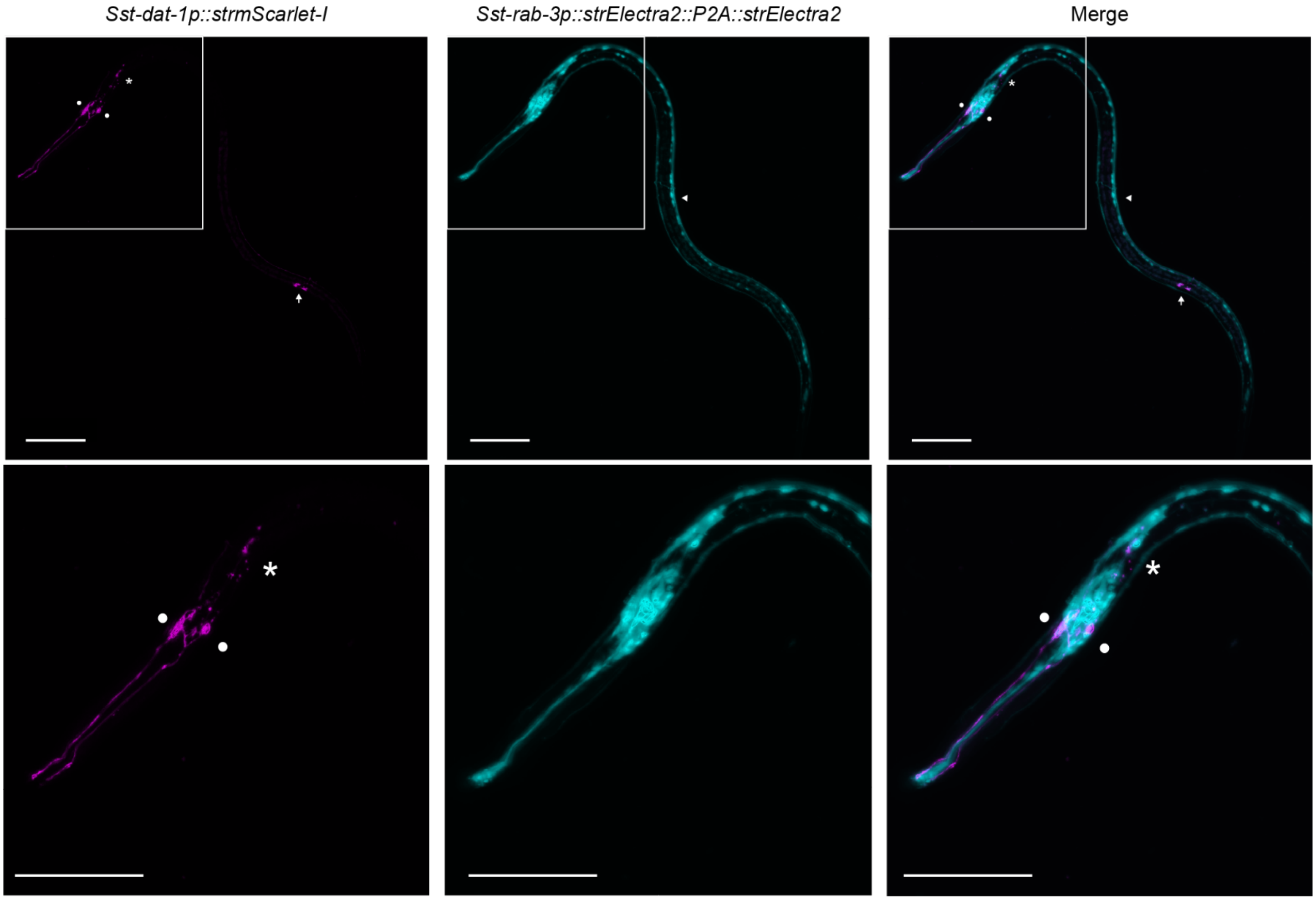

Fig. S7. Positions of the putative dopaminergic neurons in an *S. stercoralis* iL3. Montage shows the positions of the putative dopaminergic neurons, as marked by expression of the *Sst-dat-1* transcriptional reporter in magenta (left), relative to the positions of the ventral nerve cord and other neurons as marked by expression of the *Sst-rab-3* transcriptional reporter in cyan (center). Co-expression of the two reporters is shown in the merged image (right). The regions in the white boxes that show the head of the worm are enlarged in the bottom panels. The *Sst-dat-1* transcriptional reporter is as described in Figure 5B. The *Sst-rab-3* transcriptional reporter comprises a 3000 bp region upstream of the *Sst-rab-3* start codon fused with two copies of *strElectra2* separated by *P2A*, which encodes the self-cleaving peptide P2A. As described earlier, *str* indicates that the gene sequences were codon-optimized for expression in *Strongyloides* species. The circles, asterisk, and arrow label the putative *Sst*-CEP, *Sst*-ADE, and *Sst*-PDE neurons, respectively. The white arrowhead labels the ventral nerve cord. Dorsal is down and head is to the left. Scale bar = 50 µm. Source data are provided in the Source Data file.

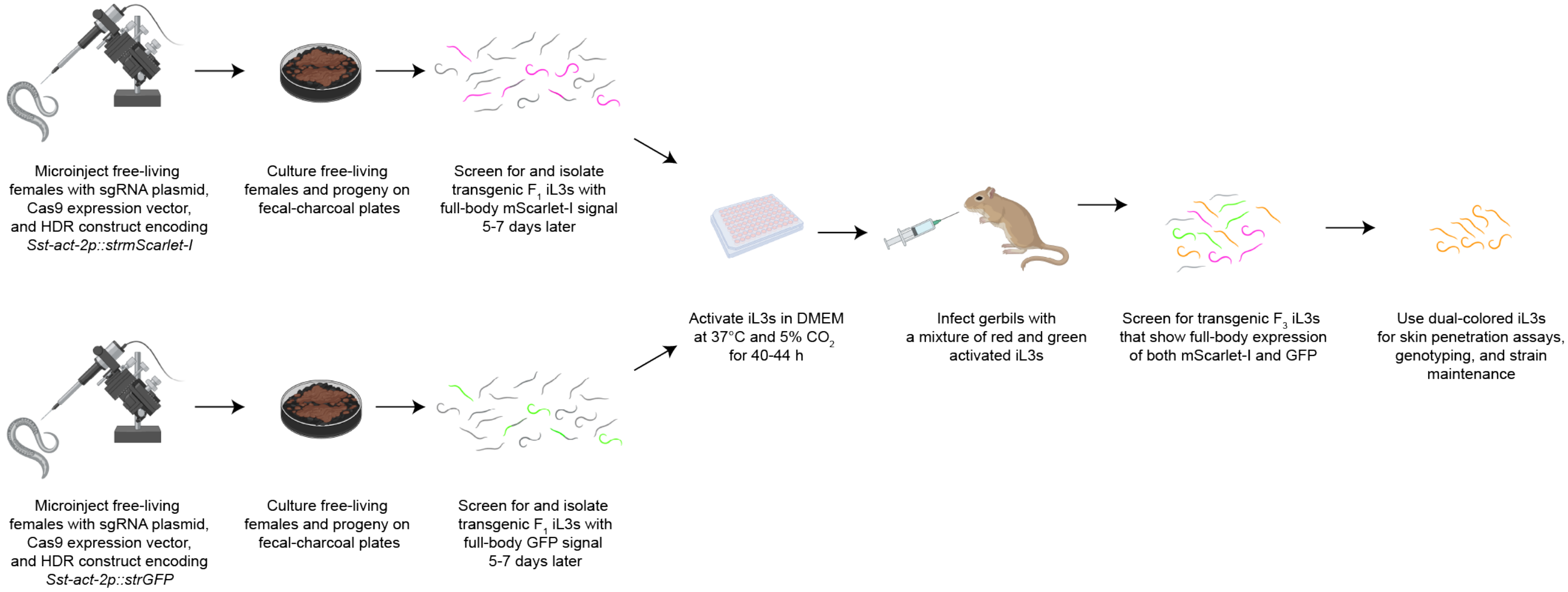

Fig. S8. Approach for generating a stable *Sst-cat-2*$^{-/-}$ line. Schematic shows the approach used to generate a mutant *Sst-cat-2*$^{-/-}$ stable line[14]. *S. stercoralis* free-living females were microinjected with one of the following mixtures: mixture 1, which consists of the Cas9 expression vector, the single guide RNA (sgRNA) expression vector, and a plasmid that provides the template for insertion of *Sst-act-2p::strmScarlet-I* into the *Sst-cat-2* locus via homology-directed repair (HDR) (top left); or mixture 2, which consists of all the components in mixture 1, except that the HDR template had the *Sst-act-2p::strGFP* transgene (bottom left). Between 5-7 days later, transgenic F_1 iL3s that expressed either mScarlet-I or GFP throughout the entire body wall muscle were selected by fluorescence microscopy; iL3s with full-body expression of either fluorescent protein are more likely to have genomic integration of the transgenes and stable transmission of the mutant allele to progeny[14,15]. These iL3s were activated by incubating them in a mixture of DMEM and antibiotics at 37°C and 5% CO_2 for 40-44 h[16-19]. The activated, transgenic iL3s were then inoculated into a single gerbil by oral gavage[7,14]. Three weeks later, after the worms cycled through a free-living generation, F_3 iL3s that expressed both mScarlet-I and GFP were selected by fluorescence microscopy. These dual-colored iL3s had one copy of the *Sst-cat-2* gene disrupted by insertion of *Sst-act-2p::strmScarlet-I* and the other copy disrupted by insertion of *Sst-act-2p::strGFP*[14]; thus, they were homozygous *Sst-cat-2*$^{-/-}$ mutants. These homozygous mutants were then used for skin-penetration assays and propagation of the strain. Created in BioRender. Mushtaqh Ali, R. (2025) https://BioRender.com/cw5cva5

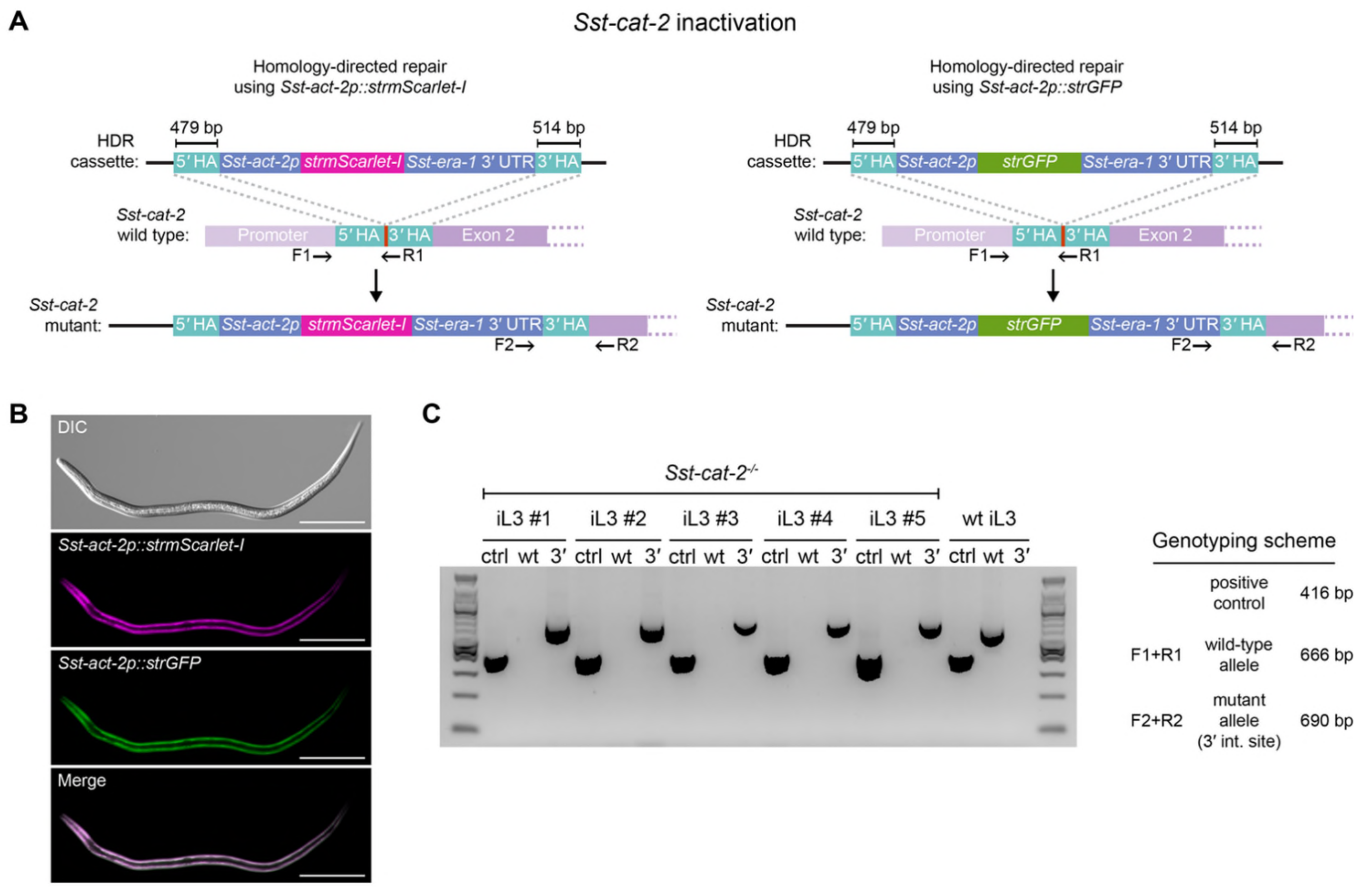

Fig. S9. Isolation of *Sst-cat-2*$^{-/-}$ iL3s. **A.** *Sst-cat-2* inactivation was achieved by integration of an *Sst-act-2* transcriptional reporter at cut sites generated by Cas9. The *Sst-act-2* transcriptional reporter consisted of the *Sst-act-2* promoter fused with a *Strongyloides*-codon-optimized gene encoding either mScarlet-I (left) or GFP (right) and the *Sst-era-1* 3′ UTR. This transgene was flanked by 5′ and 3′ homology arms (HAs); the 5′ HA matched the 479 bp fragment immediately upstream of the CRISPR site in *Sst-cat-2* (red) and the 3′ HA matched a 514 bp fragment immediately downstream of this same site. Homology-directed repair resulted in insertion of either transgene into the *Sst-cat-2* locus, creating a stop codon early in the second exon, thereby preventing expression of *Sst*-CAT-2. The approximate binding sites of genotyping primers are shown. Detection of a PCR amplicon from primers F1 and R1 indicated a wild-type locus, as R1 overlaps the Cas9 cut site. Detection of a PCR amplicon from primers F2 and R2 indicated a mutant locus, as F2 lies in the *Sst-era-1* 3′ UTR and R2 lies in the region of the *Sst-cat-2* gene that is downstream of the 3′ HA. **B.** Expression of the *Sst-act-2p::strmScarlet-I* transgene (magenta) and *Sst-act-2p::strGFP* transgene (green) across the body wall muscle of a dual-colored *Sst-cat-2*$^{-/-}$ mutant. Dorsal is up and head is left. Scale bar = 100 µm. **C.** Image shows a representative agarose gel that contains PCR amplicons from genotyping five *Sst-cat-2*$^{-/-}$ dual-colored iL3s and one wild-type iL3. For each worm, the following PCR products were loaded in order: a positive control (exon 1 of the *Sst-act-2* gene), which produced a 416 bp band (ctrl); a PCR reaction for genotyping the wild-type *Sst-cat-2* allele (primers F1 and R1), which produced a 666 bp band (wt) in wild-type iL3s; and a PCR reaction for genotyping the 3′ integration site of the HDR template (primers F2 and R2), which produced a 690 bp band (3′) in *Sst-cat-2* mutants. The first and last lanes carried a New England Biolabs 100 bp ladder. “3′ int. site” = 3′ integration site. Source data are provided in the Source Data file.

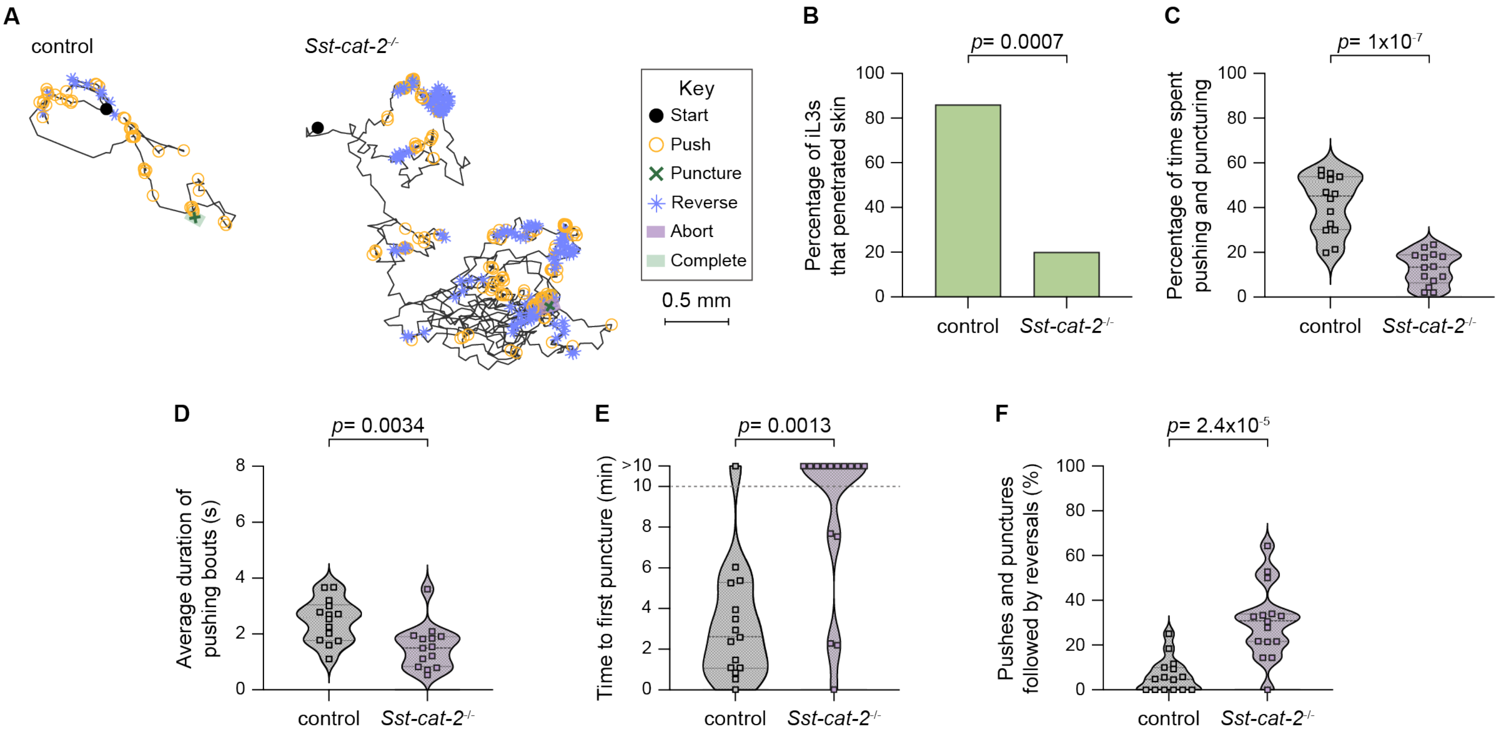

Fig. S10. Inactivation of *Sst-cat-2* severely impairs penetration of human skin and alters skin-penetration behavior. A. *Sst-cat-2$^{-/-}$* iL3s exhibit altered behaviors on human skin. Tracks show the behaviors of a representative wild-type iL3 and a representative *Sst-cat-2$^{-/-}$* iL3 on human skin. Key details behavioral motifs that were tracked. **B.** Bar graph shows the percentage of wild-type and *Sst-cat-2$^{-/-}$* iL3s that completed skin penetration. n = 15 iL3s per genotype. There are no error bars in the bar graph because the graph shows the percentage of iL3s of each genotype that completed penetration out of the total tested. **C.** Violin plot depicts the percentage of time on skin that control and *Sst-cat-2$^{-/-}$* iL3s spent engaging in pushes or punctures. n = 14 iL3s per genotype. **D.** Violin plot shows the average duration of pushing bouts for control and *Sst-cat-2$^{-/-}$* iL3s. n = 14 iL3s per genotype. **E.** Violin plot depicts the time taken by control vs. *Sst-cat-2$^{-/-}$* worms to first puncture skin. n = 15 iL3s per genotype. The dotted line at y = 10 indicates the time at which the assay ended; the dots above this line indicate animals that failed to puncture. **F.** Violin plot shows the percentage of pushes or punctures that were followed by backward locomotion that lasted at least 1 s for each genotype. n = 15 iL3s per genotype. For C-F, dots depict individual worms, dashed lines indicate medians, and dotted lines indicate interquartile ranges. Behavioral parameters plotted in B-F were obtained from 3 independent replicates, each using skin from a distinct human donor. A two-tailed Fisher's exact test was used for B, two-tailed unpaired t-tests were used for C-D, and two-tailed Mann-Whitney tests were used for E-F. Source data are provided in the Source Data file.

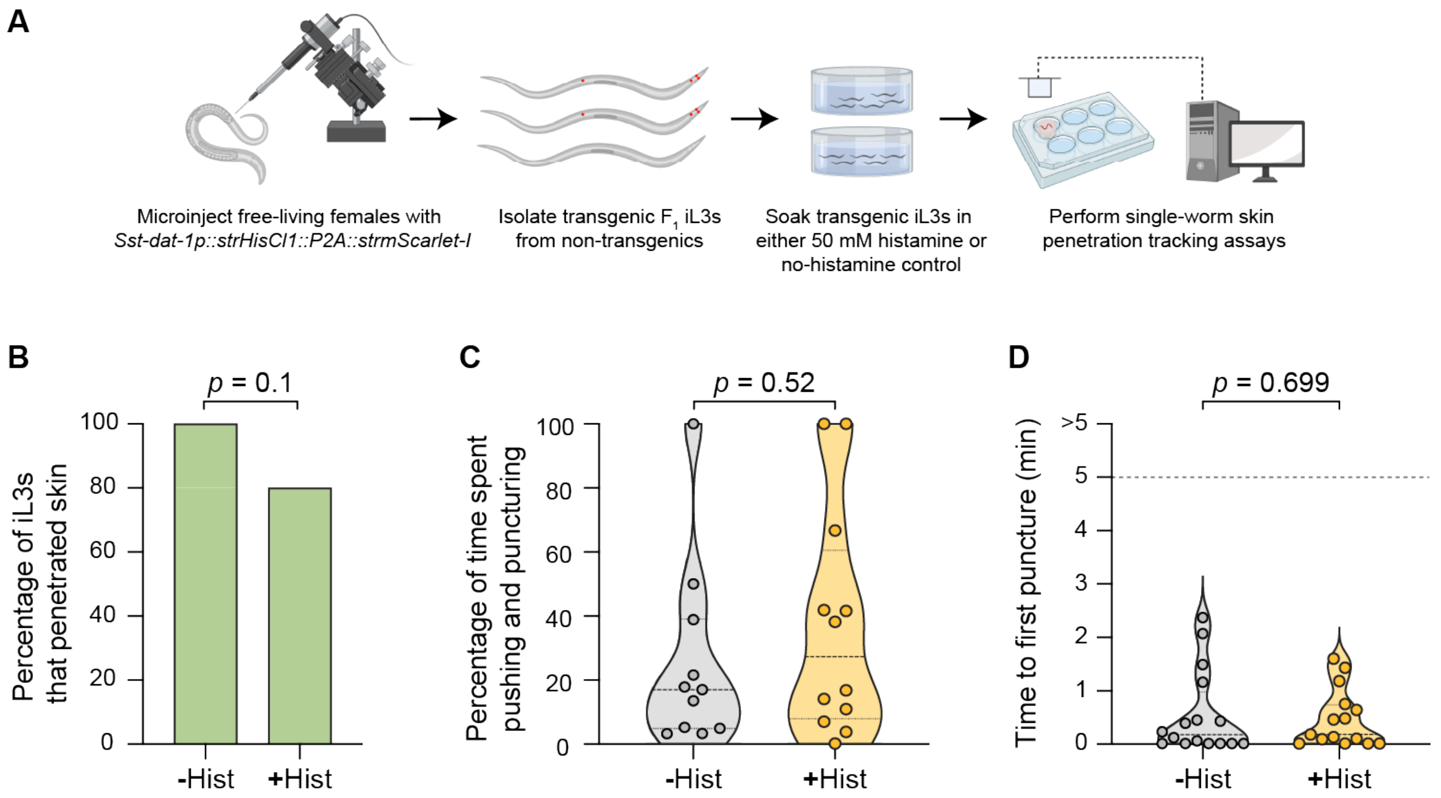

Fig. S11. Chemogenetic silencing of the dopaminergic neurons using the histamine-gated chloride channel HisCl1. A. Schematic of the approach for silencing the *S. stercoralis* dopaminergic neurons using HisCl1[20,21]. The gonads of free-living adult females were microinjected with an *Sst-dat-1p::strHisCl1::P2A::strmScarlet-I* transgene using standard techniques[22]. Transgenic F_1 iL3s that expressed both HisCl1 and mScarlet-I in the dopaminergic neurons were isolated by performing fluorescence microscopy-based screening for mScarlet-I signal. The transgenics were then separated into two groups: one group was treated with 50 mM histamine and the other group was treated with the vehicle only (ddH_2O). Skin-penetration assays were then performed as detailed in Fig. 1A. **B.** Non-transgenic wild-type iL3s exposed to either histamine (+His) or a no-histamine control (-His) penetrated rat skin similarly. Bar graph shows the percentage of -His and +His wild-type iL3s that completed skin penetration. n = 16 mock-treated and 15 histamine-treated iL3s. There are no error bars in the bar graph because the graph shows the percentage of iL3s in each treatment group that completed penetration out of the total tested. **C.** Histamine exposure does not affect pushes and punctures. Violin plot depicts the percentage of time on skin that -His vs. +His wild-type iL3s spent engaging in pushes or punctures. n = 11 -Hist and 12 +His iL3s. **D.** Histamine treatment does not affect the time to first puncture. Violin plot shows the time taken by -His vs. +His wild-type iL3s to first puncture the skin. n = 16 +His iL3s and 15 -His iL3s. The dotted line at y = 5 indicates the time at which the assay ended. For C-D, dots depict individual worms, dashed lines indicate medians, and dotted lines indicate interquartile ranges. Behavioral parameters plotted in B-D were obtained from 3 independent replicates. A two-tailed Fisher's exact test was used in B, and two-tailed Mann-Whitney tests were used in C-D. Images in A were created in BioRender. Mushtaqh Ali, R. (2025) https://BioRender.com/pxiaiyq. Source data are provided in the Source Data file.

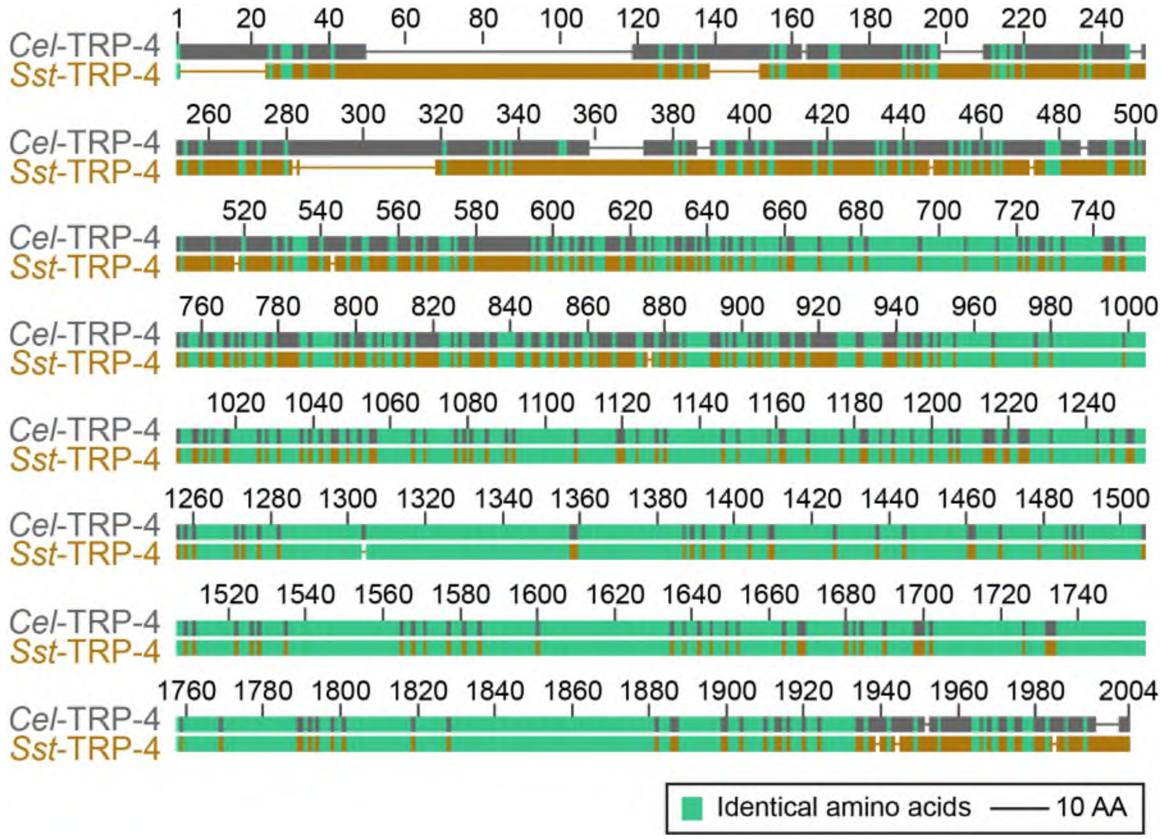

Fig. S12. The *S. stercoralis* homolog of *Cel*-TRP-4. Schematic representation of the alignment of the amino acid sequences of the *C. elegans* TRP-4 protein (top) and the *S. stercoralis* TRP-4 protein (bottom). The amino acid sequences of the two proteins are 57.1% identical. Identical amino acids are depicted in green. Drawings are to scale and the scale bar = 10 amino acids (AA).

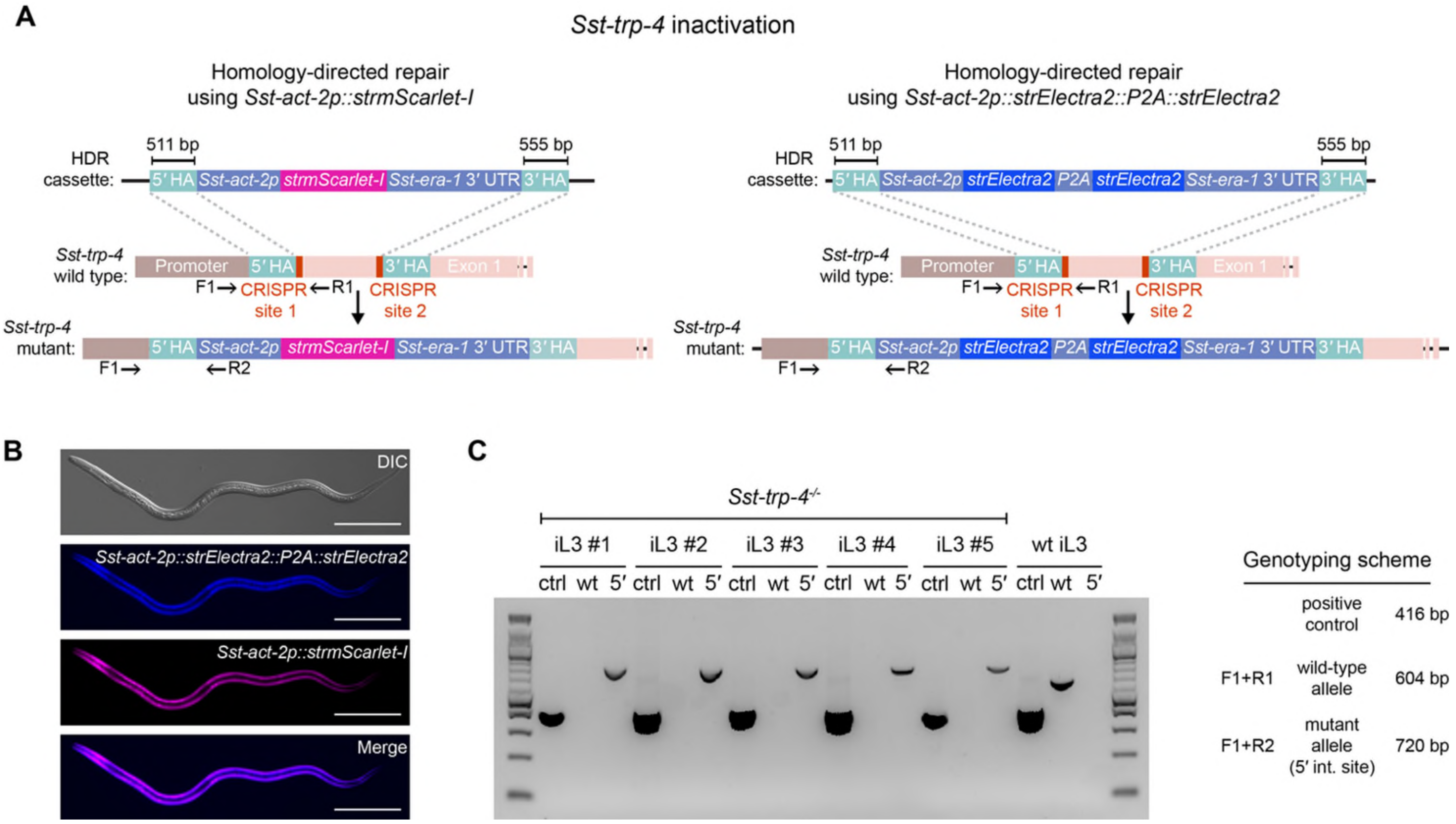

Fig. S13. Isolation of *Sst-trp-4*$^{-/-}$ iL3s. A. *Sst-trp-4* inactivation was achieved by integration of an *Sst-act-2* transcriptional reporter at cut sites generated by Cas9. The overall principle and approach for generating *Sst-trp-4* knockouts was similar to that detailed in Fig. S9 with the following differences: 1) two distinct sgRNAs, targeting CRISPR sites 1 and 2 (red), were used in conjunction with Cas9 to generate double-strand breaks; 2) repair templates containing *Sst-act-2p::strmScarlet-I* (left) and *Sst-act-2p::strElectra2::P2A::strElectra2*[23] (right) were used for homology-directed repair; and 3) the 5′ homology arm matched a 511 bp fragment upstream of CRISPR site 1 and the 3′ HA matched a 555 bp fragment downstream of CRISPR site 2. Approximate binding sites of genotyping primers for detection of wild-type and mutant *Sst-trp-4* alleles are shown. Detection of PCR amplicons from F1 and R1 indicated a wild-type locus, as R1 overlaps the region that is excised by Cas9. Detection of PCR amplicons from F1 and R2 indicated a mutant locus, as F1 lies in the region of the *Sst-trp-4* promoter that is upstream of the 5′ HA and R2 lies in the *Sst-act-2* promoter. **B.** Expression of the *Sst-act-2p::strElectra2::P2A::strElectra2::Sst-era-1* 3′ UTR (blue) and *Sst-act-2p::strmScarlet-I::Sst-era-1* 3′ UTR (magenta) transgenes across the body wall muscle of a dual-colored *Sst-trp-4*$^{-/-}$ mutant. Dorsal is up and head is left. Scale bar = 100 µm. **C.** Image shows an agarose gel that contains amplicons from genotyping PCRs of five *Sst-trp-4*$^{-/-}$ dual-colored iL3s and one wild-type iL3. For each worm, the following PCR products were loaded in order: a positive control (same as Fig. S9); a PCR reaction for detecting the wild-type *Sst-trp-4* allele (primers F1 and R1), which produced a 604 bp band (wt) in wild-type iL3s; and a PCR reaction for genotyping the 5′ integration site of the HDR template (primers F1 and R2), which produced a 720 bp band (5′) in carriers of the mutant *Sst-trp-4* allele. The first and last lanes carried a New England Biolabs 100 bp ladder. “5′ int. site” = 5′ integration site. Source data are provided in the Source Data file.

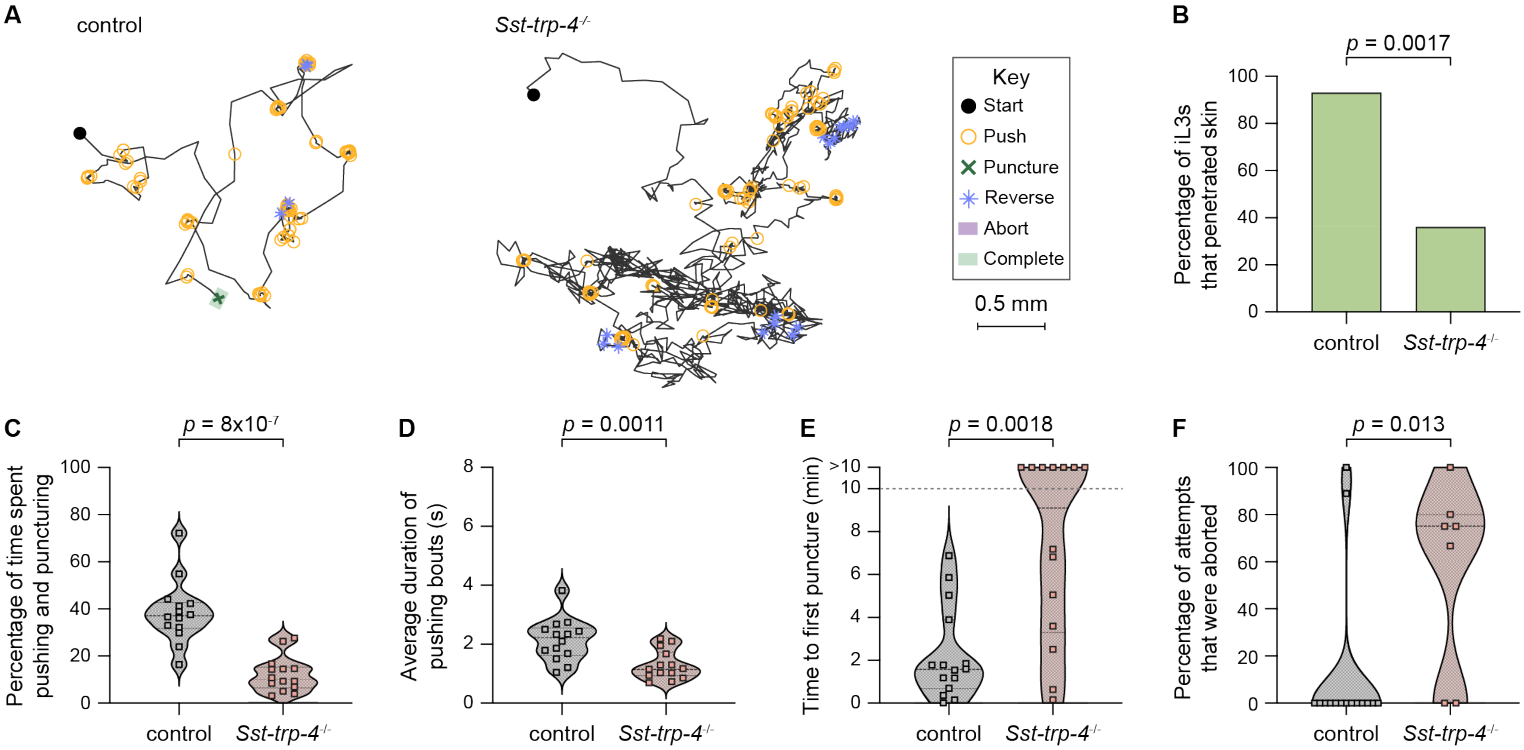

Fig. S14. Inactivation of *Sst-trp-4* severely impairs the ability to penetrate human skin. A. *Sst-trp-4*$^{-/-}$ iL3s exhibit reduced skin penetration and altered behaviors on human skin. Tracks show the behaviors of a representative wild-type iL3 and a representative *Sst-trp-4*$^{-/-}$ iL3. Key details the behavioral motifs that were tracked. **B.** *Sst-trp-4*$^{-/-}$ iL3s have severely reduced skin-penetration ability. Bar graph shows the percentage of control and *Sst-trp-4*$^{-/-}$ iL3s that completed skin penetration. n = 15 control and 14 *Sst-trp-4*$^{-/-}$ iL3s. There are no error bars in the bar graph because the graph shows the percentage of iL3s of each genotype that completed penetration out of the total number tested. **C.** Violin plot depicts the percentage of time on skin that control and *Sst-trp-4*$^{-/-}$ iL3s spent engaging in pushes or punctures. n = 14 iL3s per genotype. **D.** Violin plot shows the average duration of pushing bouts for control and *Sst-trp-4*$^{-/-}$ iL3s. n = 14 iL3s per genotype. **E.** Violin plot depicts the time taken by control vs. *Sst-trp-4*$^{-/-}$ iL3s to first puncture skin. n = 14 iL3s per genotype. The dotted line at y = 10 indicates the time at which the assay ended; dots above this line indicate animals that failed to puncture the skin. **F.** Violin plot depicts the percentage of penetration attempts, as defined by instances that the worm punctured and partially entered the skin, that were aborted. n = 15 control and 7 *Sst-trp-4*$^{-/-}$ iL3s. For C-F, dots depict individual worms, dashed lines indicate medians, and dotted lines indicate interquartile ranges. Behavioral parameters plotted in B-F were obtained from 3 independent replicates, each using skin from a distinct human donor. A two-tailed Fisher's exact test was used for B, two-tailed unpaired t-tests were used for C-D, and two-tailed Mann-Whitney tests were used for E-F. Source data are provided in the Source Data file.

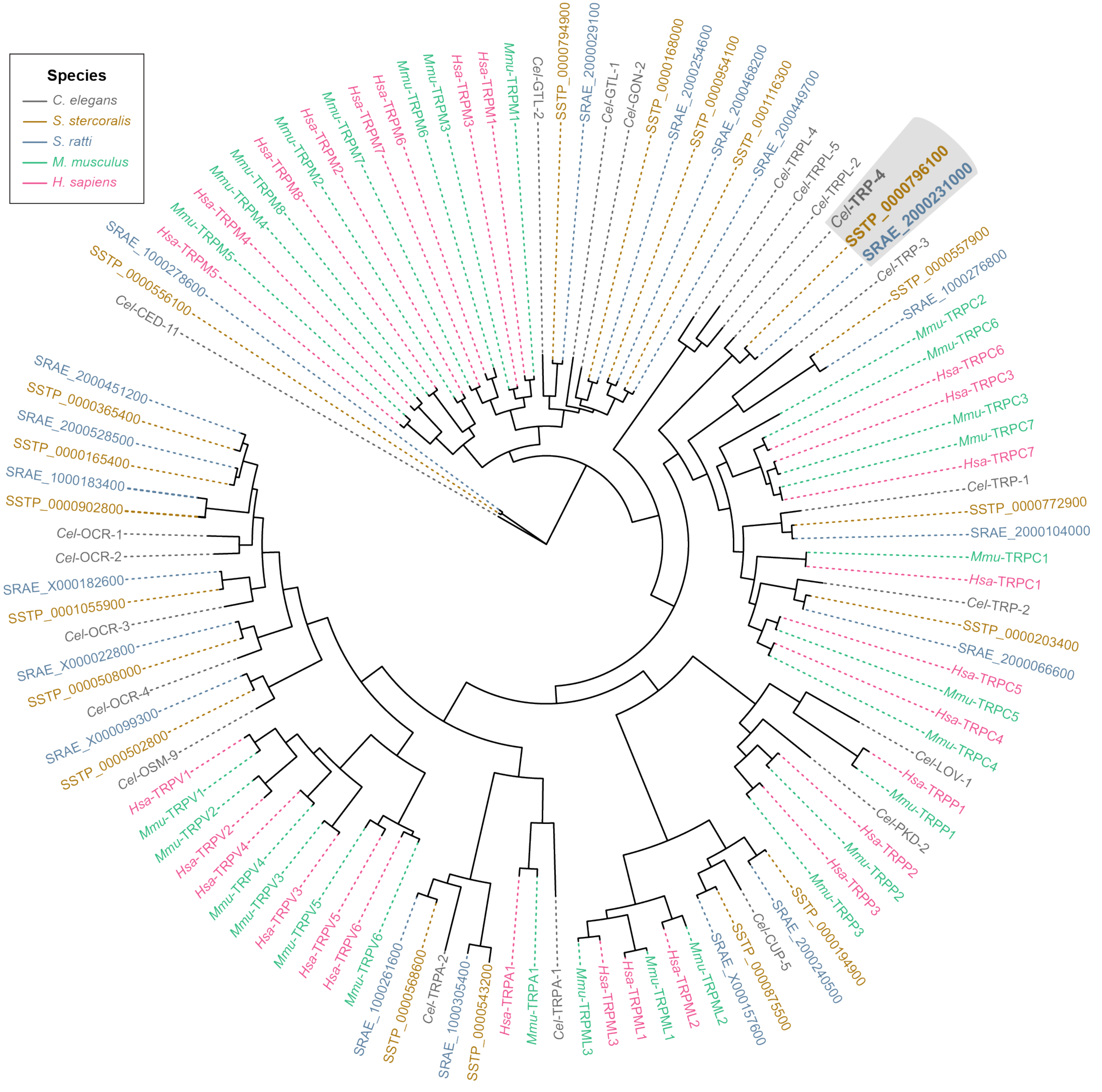

Fig. S15. *Sst*-TRP-4 is not conserved to humans. Phylogenetic analysis of the TRP family members of *C. elegans* (gray), *S. stercoralis* (brown), *S. ratti* (blue), *Mus musculus* (green) and *Homo sapiens* (pink). The putative TRP family members of *S. stercoralis* and *S. ratti* were identified by performing TBLASTN searches of the *C. elegans* TRP channels[24-26] against either the *S. stercoralis* genome or the *S. ratti* genome in WBPS18. The sequences of known *M. musculus* and *H. sapiens* TRP channels[27] were retrieved from UniProtKB (Release 2024_06); the related accession numbers are listed in Table S2.

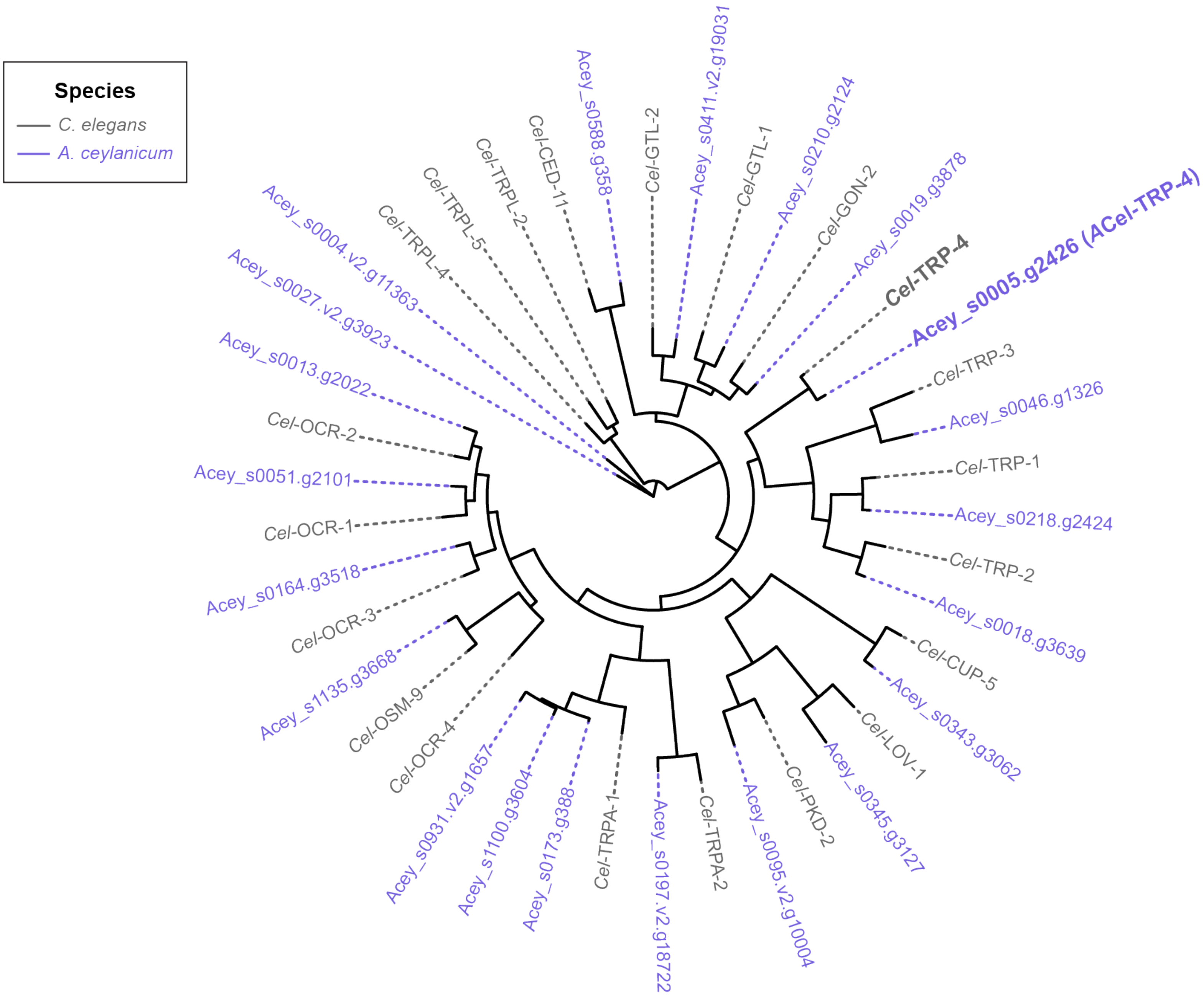

Fig. S16. *Sst*-TRP-4 is conserved to hookworms. Phylogenetic analysis of the TRP family members of *C. elegans* (gray) and *A. ceylanicum* (purple). The putative TRP family members of *A. ceylanicum* were identified by performing TBLASTN searches of the *C. elegans* TRP channels[24-26] against the *A. ceylanicum* genome (PRJNA23179) in WBPS19.

Table S1. Oligonucleotides used for construct generation and genotyping worms from CRISPR/Cas9 experiments.

Primer name	Sequence	Description/Use
SG78	5′-GTATTCCCTTCTATTGTTGGAAGACC-3′	Forward primer for genotyping *Sst-act-2* exon 1; positive control
SG80	5′-CCTTCATAGATTGGTACAGTGTGAG-3′	Reverse primer for genotyping *Sst-act-2* exon 1; positive control
RP32	5′-CGTAATTAAAAATATTTAAAAGTTACTATTACG-3′	Forward primer in *Sst-cat-2* promoter for genotyping wild-type *Sst-cat-2* allele; “Primer F1”
RP33	5′-GGTATCTAATTTTGCTAATGGTGG-3′	Reverse primer in *Sst-cat-2* exon 2 (overlapping CRISPR site) for genotyping wild-type *Sst-cat-2* allele; “Primer R1”
RP30	5′-CGGTATATTTTACTTCAATGTGG-3′	Forward primer in *Sst-era-1* 3′ UTR for genotyping *Sst-cat-2* 3′ integration band; “Primer F2”
RP34	5′-TTAGCCATTTTATTTAACTCTTCACG-3′	Reverse primer in *Sst-cat-2* exon 2 for genotyping *Sst-cat-2* 3′ integration band; “Primer R2”
RP39	5′-ATTTGGATGAAGACTTTTTCATACC-3′	Forward primer in *Sst-trp-4* promoter for genotyping wild-type *Sst-trp-4* allele and 5′ integration band; “Primer F1”
RP46	5′-AATGATGACCATATTGCTCCAGC-3′	Reverse primer in *Sst-trp-4* exon 1 for genotyping wild-type *Sst-trp-4* allele; “Primer R1”
RP28	5′-CGAGGAACCTCTTTTCCACAC-3′	Reverse primer in *Sst-act-2* promoter for genotyping *Sst-trp-4* 5′ integration band; “Primer R2”
RP15	5′-TCAAGCTTTTTATTATAATTTTTTTTTATTTCAAT TATATA-3′	Forward primer for amplifying the promoter region of *Sst-dat-1*
RP16	5′-ATACCGGTTTGATTTTAAAAATCTTTTATTAATG ATTC-3′	Reverse primer for amplifying the promoter region of *Sst-dat-1*

Table S2. UniProtKB accession numbers for *M. musculus* and *H. sapiens* TRP channels.

Species	Gene Name	UniProtKB accession number		Gene Name	UniProtKB accession number
Homo sapiens	TRPV1	Q8NER1		TRPC3	Q13507
	TRPV2	Q9Y5S1		TRPC4	Q9UBN4
	TRPV3	Q8NET8		TRPC5	Q9UL62
	TRPV4	Q9HBA0		TRPC6	Q9Y210
	TRPV5	Q9NQA5		TRPC7	Q9HCX4
	TRPV6	Q9H1D0		TRPM1	Q7Z4N2
	TRPA1	O75762		TRPM2	O94759
	TRPML1	Q9GZU1		TRPM3	Q9HCF6
	TRPML2	Q8IZK6		TRPM4	Q8TD43
	TRPML3	Q8TDD5		TRPM5	Q9NZQ8
	TRPP1	P98161		TRPM6	Q9BX84
	TRPP2	Q13563		TRPM7	Q96QT4
	TRPP3	Q9P0L9		TRPM8	Q7Z2W7
	TRPC1	P48995			
Mus musculus	TRPV1	Q704Y3		TRPC2	Q9R244
	TRPV2	Q9WTR1		TRPC3	Q9QZC1
	TRPV3	Q8K424		TRPC4	Q9QUQ5
	TRPV4	Q9EPK8		TRPC5	Q9QX29
	TRPV5	P69744		TRPC6	Q61143
	TRPV6	Q91WD2		TRPC7	Q9WVC5
	TRPA1	Q8BLA8		TRPM1	Q2TV84
	TRPML1	Q99J21		TRPM2	Q91YD4
	TRPML2	Q8K595		TRPM3	J9SQF3
	TRPML3	Q8R4F0		TRPM4	Q7TN37
	TRPP1	O08852		TRPM5	Q9JJH7
	TRPP2	O35245		TRPM6	Q8CIR4
	TRPP3	A2A259		TRPM7	Q923J1
	TRPC1	Q61056		TRPM8	Q8R4D5

SUPPLEMENTAL REFERENCES

1 Viney, M. E. & Lok, J. B. The biology of *Strongyloides* spp. In *WormBook,* www.wormbook.org, p. 1-17 (2015).

2 Noskova, E. *et al. Strongyloides* in non-human primates: significance for public health control. *Philos Trans R Soc Lond B Biol Sci* **379**, 20230006, doi:10.1098/rstb.2023.0006 (2024).

3 Jaleta, T. G. *et al.* Different but overlapping populations of *Strongyloides stercoralis* in dogs and humans – dogs as a possible source for zoonotic strongyloidiasis. *PLoS Negl Trop Dis* **11**, e0005752, doi:10.1371/journal.pntd.0005752 (2017).

4 Genta, R. M. & Caymmi Gomes, M. Pathology. In *Strongyloidiasis: a major roundworm infection of man* (ed D.I. Grove) 105-132 (Taylor & Francis, Ltd., 1989).

5 Dionisio, D. *et al. Strongyloides stercoralis*: ultrastructural study of newly hatched larvae within human duodenal mucosa. *J Clin Pathol* **53**, 110-116, doi:10.1136/jcp.53.2.110 (2000).

6 Mendez, P., Walsh, B. & Hallem, E. A. Using newly optimized genetic tools to probe *Strongyloides* sensory behaviors. *Mol Biochem Parasitol* **250**, 111491, doi:10.1016/j.molbiopara.2022.111491 (2022).

7 Patel, R. *et al.* The generation of stable transgenic lines in the human-infective nematode *Strongyloides stercoralis*. *G3* **14**, jkae122, doi:10.1093/g3journal/jkae122 (2024).

8 Gordon, C. A. *et al.* Strongyloidiasis. *Nat Rev Dis Primers* **10**, 6, doi:10.1038/s41572-023-00490-x (2024).

9 Czeresnia, J. M. & Weiss, L. M. *Strongyloides stercoralis*. *Lung* **200**, 141-148, doi:10.1007/s00408-022-00528-z (2022).

10 Lee, Y. & Hwang, K. Skin thickness of Korean adults. *Surg Radiol Anat* **24**, 183-189, doi:10.1007/s00276-002-0034-5 (2002).

11 Oltulu, P., Ince, B., Kokbudak, N., Findik, S. & Kilinc, F. Measurement of epidermis, dermis, and total skin thicknesses from six different body regions with a new ethical histometric technique. *Turk J Plast Surg* **26**, 56-61 (2018).

12 Fitzpatrick, P. F. The aromatic amino acid hydroxylases: structures, catalysis, and regulation of phenylalanine hydroxylase, tyrosine hydroxylase, and tryptophan hydroxylase. *Arch Biochem Biophys* **735**, 109518, doi:10.1016/j.abb.2023.109518 (2023).

13 Wang, J. *et al.* The conserved domain database in 2023. *Nucleic Acids Res* **51**, D384-D388, doi:10.1093/nar/gkac1096 (2023).

14 Banerjee, N. *et al.* Carbon dioxide shapes parasite-host interactions in a human-infective nematode. *Curr Biol* **35**, 277-286.e276, doi:10.1016/j.cub.2024.11.036 (2025).

15 Gang, S. S. *et al.* Targeted mutagenesis in a human-parasitic nematode. *PLoS Pathog* **13**, e1006675, doi:10.1371/journal.ppat.1006675 (2017).

16 Ashton, F. T., Zhu, X., Boston, R., Lok, J. B. & Schad, G. A. *Strongyloides stercoralis*: amphidial neuron pair ASJ triggers significant resumption of development by infective larvae under host-mimicking *in vitro* conditions. *Exp Parasitol* **115**, 92-97, doi:10.1016/j.exppara.2006.08.010 (2007).

17 Stoltzfus, J. D., Massey, H. C., Jr., Nolan, T. J., Griffith, S. D. & Lok, J. B. *Strongyloides stercoralis age-1*: a potential regulator of infective larval development in a parasitic nematode. *PLoS ONE* **7**, e38587, doi:10.1371/journal.pone.0038587 (2012).

18 Stoltzfus, J. D., Bart, S. M. & Lok, J. B. cGMP and NHR signaling co-regulate expression of insulin-like peptides and developmental activation of infective larvae in *Strongyloides stercoralis*. *PLoS Pathog* **10**, e1004235, doi:10.1371/journal.ppat.1004235 (2014).

19 Gang, S. S. *et al.* Chemosensory mechanisms of host seeking and infectivity in skin-penetrating nematodes. *Proc Natl Acad Sci USA* **117**, 17913-17923, doi:10.1073/pnas.1909710117 (2020).

20 Pokala, N., Liu, Q., Gordus, A. & Bargmann, C. I. Inducible and titratable silencing of *Caenorhabditis elegans* neurons *in vivo* with histamine-gated chloride channels. *Proc Natl Acad Sci USA* **111**, 2770-2775, doi:10.1073/pnas.1400615111 (2014).

21 Bryant, A. S., Ruiz, F., Lee, J. & Hallem, E. A. The neural basis of heat seeking in a human-infective parasitic worm. *Curr Biol* **32**, 2206-2221, doi:10.1101/2021.06.23.449647 (2022).

22 Castelletto, M. L. & Hallem, E. A. Generating transgenics and knockouts in *Strongyloides* species by microinjection. *J Vis Exp* **176**, e63023, doi:10.3791/63023 (2021).

23 Papadaki, S. *et al.* Dual-expression system for blue fluorescent protein optimization. *Sci Rep* **12**, 10190, doi:10.1038/s41598-022-13214-0 (2022).

24 Xiao, R. & Xu, X. Z. *C. elegans* TRP channels. *Adv Exp Med Biol* **704**, 323-339, doi:10.1007/978-94-007-0265-3_18 (2011).

25 Kahn-Kirby, A. H. & Bargmann, C. I. TRP channels in *C. elegans*. *Annu Rev Physiol* **68**, 719-736, doi:10.1146/annurev.physiol.68.040204.100715 (2006).

26 Goodman, M. B. Mechanosensation. In *WormBook,* www.wormbook.org, p. 1-14 (2006).

27 Venkatachalam, K. & Montell, C. TRP channels. *Annu Rev Biochem* **76**, 387-417, doi:10.1146/annurev.biochem.75.103004.142819 (2007).
