# Supplementary material for: Dopamine signaling drives skin invasion by human-infective nematodes
Source: Nat Commun. 2025 Aug 13;16:7246. doi: 10.1038/s41467-025-62517-z (PMC12350745; doi:10.1038/s41467-025-62517-z)
Supplement: Supplementary file 2 — Description Of Additional Supplementary File [file 41467_2025_62517_MOESM2_ESM.pdf]

Description of Additional supplementary files

Movie S1.

Skin-penetration behavior of an S. stercoralis iL3 on rat skin.

Movie shows time-lapse images of an S. stercoralis iL3 (expressing Sst-act-2p::strmScarlet-I) penetrating rat skin. Images were acquired at 2 frames/s and the playback speed is 8 frames/s. Behavioral motifs that were quantified are annotated.

Movie S2.

Skin-penetration behavior of a different S. stercoralis iL3 on rat skin.

Movie shows time-lapse images of an S. stercoralis iL3 (expressing Sst-act-2p::strmScarlet-I) penetrating rat skin. This iL3 was a wild-type transgenic F1 iL3 obtained from microinjection of free-living females with CRISPR/Cas9 components for inactivating Sst-cat-2. Images were acquired at 2 frames/s and the playback speed is 8 frames/s. Behavioral motifs that were quantified are annotated.

Movie S3.

Skin-penetration behavior of an S. ratti iL3 on rat skin.

Movie shows time-lapse images of an S. ratti iL3 (expressing Sst-act-2p::strmScarlet-I) penetrating rat skin. Images were acquired at 2 frames/s and the playback speed is 8 frames/s. Behavioral motifs that were quantified are annotated.

Movie S4.

Expression of Sst-dat-1p::strmScarlet-I and Sst-rab-3p::strElectra2::P2A::strElectra2 in an S. stercoralis iL3.

Movie shows individual images captured every 2.23 µm throughout the volume of an S. stercoralis iL3 expressing transcriptional reporters for Sst-dat-1 and Sst-rab-3. The Sst-dat-1 reporter is expressed in the putative S. stercoralis dopaminergic neurons, and the Sst-rab-3 reporter is predicted to be expressed in most S. stercoralis neurons. The putative Sst-CEP, Sst-ADE, and Sst-PDE neurons are labeled as they come into focus. Playback speed = 1 frame/s.
