# Supplementary material for: Dopamine signaling drives skin invasion by human-infective nematodes
Source: Nat Commun. 2025 Aug 13;16:7246. doi: 10.1038/s41467-025-62517-z (PMC12350745; doi:10.1038/s41467-025-62517-z)
Supplement: Supplementary file 7 — Reporting summary [file 41467_2025_62517_MOESM7_ESM.pdf]

Corresponding author(s): Elissa A. Hallem

Last updated by author(s): May 23, 2025

Reporting Summary

Nature Portfolio wishes to improve the reproducibility of the work that we publish. This form provides structure for consistency and transparency in reporting. For further information on Nature Portfolio policies, see our Editorial Policies and the Editorial Policy Checklist.

Statistics

For all statistical analyses, confirm that the following items are present in the figure legend, table legend, main text, or Methods section.

n/a	Confirmed	
☐	☒	The exact sample size (*n*) for each experimental group/condition, given as a discrete number and unit of measurement
☐	☒	A statement on whether measurements were taken from distinct samples or whether the same sample was measured repeatedly
☐	☒	The statistical test(s) used AND whether they are one- or two-sided<br>*Only common tests should be described solely by name; describe more complex techniques in the Methods section.*
☐	☒	A description of all covariates tested
☐	☒	A description of any assumptions or corrections, such as tests of normality and adjustment for multiple comparisons
☐	☒	A full description of the statistical parameters including central tendency (e.g. means) or other basic estimates (e.g. regression coefficient) AND variation (e.g. standard deviation) or associated estimates of uncertainty (e.g. confidence intervals)
☒	☐	For null hypothesis testing, the test statistic (e.g. *F*, *t*, *r*) with confidence intervals, effect sizes, degrees of freedom and *P* value noted<br>*Give P values as exact values whenever suitable.*
☒	☐	For Bayesian analysis, information on the choice of priors and Markov chain Monte Carlo settings
☒	☐	For hierarchical and complex designs, identification of the appropriate level for tests and full reporting of outcomes
☐	☒	Estimates of effect sizes (e.g. Cohen's *d*, Pearson's *r*), indicating how they were calculated

Our web collection on statistics for biologists contains articles on many of the points above.

Software and code

Policy information about availability of computer code

Data collection	Custom code used for skin- penetration tracking assays is available from GitHub (https://github.com/BryantLabUW/WormTracker3000.git).
Data analysis	Statistical analyses were performed in Prism 10.0.0. Sample sizes were determined by power analysis using G*Power 3.1.9.6.

For manuscripts utilizing custom algorithms or software that are central to the research but not yet described in published literature, software must be made available to editors and reviewers. We strongly encourage code deposition in a community repository (e.g. GitHub). See the Nature Portfolio guidelines for submitting code & software for further information.

Data

Policy information about availability of data

All manuscripts must include a data availability statement. This statement should provide the following information, where applicable:

- Accession codes, unique identifiers, or web links for publicly available datasets
- A description of any restrictions on data availability
- For clinical datasets or third party data, please ensure that the statement adheres to our policy

All data necessary for the conclusions described in this study are included with this article. Raw data from all figures can be found in the Source Data file. The behavior tracking files and RNA-Seq data for Sst-cat-2, Sst-dat-1, and Sst-trp-4 are available from GitHub (https://github.com/HallemLab/Patel_et_al_2025). The raw images of behavior and expression profiles of Sst-cat-2, Sst-dat-1, Sst-trp-4, and Sst-rab-3 are available on BioImage Archive (https://www.ebi.ac.uk/biostudies/bioimages/studies/S-BIAD1970).

Research involving human participants, their data, or biological material

Policy information about studies with human participants or human data. See also policy information about sex, gender (identity/presentation), and sexual orientation and race, ethnicity and racism.

Reporting on sex and gender	Human skin samples were obtained based on availability, regardless of sex or gender. Skin from both male and female donors was used.
Reporting on race, ethnicity, or other socially relevant groupings	No socially relevant groupings were included in the manuscript.
Population characteristics	Samples were obtained either commercially from a cadaver donor (via Accio BioBank) or from patients of the UCLA Dermatology Clinic.
Recruitment	Skin samples were collected from adult patients (30-50 years old, either male or female) who were undergoing elective plastic surgery at the UCLA Dermatology Clinic.
Ethics oversight	Human skin samples were collected following approval by the University of California Institutional Review Board (Protocol 22-000400), with signed written informed consent obtained in accordance with the Declaration of Helsinki principles.

Note that full information on the approval of the study protocol must also be provided in the manuscript.

Field-specific reporting

Please select the one below that is the best fit for your research. If you are not sure, read the appropriate sections before making your selection.

☒ Life sciences ☐ Behavioural & social sciences ☐ Ecological, evolutionary & environmental sciences

For a reference copy of the document with all sections, see nature.com/documents/nr-reporting-summary-flat.pdf

Life sciences study design

All studies must disclose on these points even when the disclosure is negative.

Sample size

Sample sizes were determined by power analysis using G*Power 3.1.9.6.

Data exclusions

Skin-penetration assays:

1. For all assays, iL3s that had initiated penetration by the time the recording started were excluded from this analysis. This exclusion criteria was established in advance because we could not accurately track the behavior of these iL3s from the time of placement on the skin.

2. For assays with rat skin, iL3s were excluded if they were not clearly visible on the skin surface because of crawling for 10 s or more in a portion of the skin that had a lot of fur stuck to it. iL3s were also excluded if they got trapped under a portion of fur and could not move out from underneath it.

3. For assays with both rat and human skin, iL3s were excluded if the time to puncture could not be accurately ascertained because of the posture of the iL3 on the skin. Sometimes the nose was difficult to see, in which case it was not possible to tell when the nose was outside the skin and when it was inside the skin. In addition, iL3s were excluded if they crossed paths with an iL3 from a previous assay on the same skin piece if it could not be ascertained which iL3 was the one originally being filmed and which one was the one that crawled into the field of view from a prior assay.

4. For assays with Ancylostoma ceylanicum, iL3s were often not visible or faintly visible because DiI did not properly stain these worms. Thus, if any iL3s could not be seen properly during the recording, the recording was stopped and the worm was excluded from the analysis. In addition, if an iL3 was recorded for the full 5-minute assay period but could not be seen for 10 s or more whilst performing the data analysis, it was excluded.

5. For assays with human skin, iL3s were excluded if they were either not visible for a total time of 2 minutes or more due to being in a divot on the skin surface, or if they crawled more than ~1 cm away from the exfoliated portion of the skin.

6. For all assays, iL3s were excluded from the analysis if they looked damaged upon transfer to the skin and/or did not crawl off the paintbrush while being transferred onto the assay surface.

7. For assays with DiI-stained worms, iL3s were excluded if they looked like they were trying to crawl from the drop-off point but appeared stuck to the skin surface. In some cases, the DiI or dimethylformamide solvent appeared to make the outside of the worm sticky, which prevented the worms from moving on the skin surface.

Movement-tracking assays:

1. For movement-tracking assays with DiI-stained worms, iL3s were excluded if they looked like they were trying to crawl from the drop-off point but appeared stuck to the plate surface. The reason is as described above (see point 7).

Replication	All experimental results were replicated across multiple days and with distinct populations of worms and skin donors. All experimental trials supported our results.
Randomization	For the assays with blinding (see below), each condition was tested on the same piece of skin and the order of testing was switched between one piece of skin and the next. For example, if 5 worms from condition A were tested first and then 5 worms from condition B were tested second on skin piece 1, then on skin piece 2, 5 worms of condition B were tested first and then 5 worms from condition A.<br><br>For assays with haloperidol, each treatment group was tested in a randomized order. In addition, the order of testing of treatment groups was switched from one biological replicate to the next. Similarly, for assays examining the behavior on host vs non-host skin, the order of testing between human skin and rat skin was switched from one biological replicate to the next; in some replicates, human skin was tested first, in others rat skin was tested first.
Blinding	All assays were done blind to genotype or treatment condition, except for comparisons of worm behavior on host vs. non-host skin and haloperidol assays. Blinding was lifted after the experiment was over.

Reporting for specific materials, systems and methods

We require information from authors about some types of materials, experimental systems and methods used in many studies. Here, indicate whether each material, system or method listed is relevant to your study. If you are not sure if a list item applies to your research, read the appropriate section before selecting a response.

Materials & experimental systems

n/a	Involved in the study
☒	☐ Antibodies
☒	☐ Eukaryotic cell lines
☒	☐ Palaeontology and archaeology
☐	☒ Animals and other organisms
☒	☐ Clinical data
☒	☐ Dual use research of concern
☒	☐ Plants

Methods

n/a	Involved in the study
☒	☐ ChIP-seq
☒	☐ Flow cytometry
☒	☐ MRI-based neuroimaging

Animals and other research organisms

Policy information about studies involving animals; ARRIVE guidelines recommended for reporting animal research, and Sex and Gender in Research

Laboratory animals	For maintenance of S. stercoralis infections, Mongolian gerbils (Meriones unguiculatus Strain 243, Charles River Laboratories) aged ~1.5-3 months were used. For maintenance of S. ratti infections, outbred Sprague-Dawley rats (Rattus norvegicus Hsd:Sprague Dawley® SD®, Inotiv) aged ~1.5-4 months were used. For maintenance of Ancylostoma ceylanicum, Golden Syrian hamsters (Mesocricetus auratus HsdHan®:AURA, Inotiv) aged >5 weeks were used. For ex vivo skin-penetration assays, outbred Sprague-Dawley rats (Rattus norvegicus Hsd:Sprague Dawley® SD®, Inotiv) aged 3-13 months were used. For in vivo skin-penetration assays, outbred Sprague-Dawley rats (Rattus norvegicus Hsd:Sprague Dawley® SD®, Inotiv) aged 8-10 months were used.
Wild animals	N/A
Reporting on sex	Ex vivo and in vivo skin-penetration assays were performed using skin from male and female rats. No differences in nematode behavior were observed on skin from male vs. female rats. Thus, skin from either male or female rats was used, depending on availability. Because no differences in behavior were observed on male vs. female rat skin, and because the experimental groups were nematodes rather than rats, the data from different skin donors were pooled. Rat skin donors as as follows: all rats used in ex vivo skin-penetration assays were outbred Sprague-Dawley rats (Rattus norvegicus Hsd:Sprague Dawley® SD®, Inotiv). Three 10-month-old male rats were used for skin-penetration assays in Fig. 2A-D. An 8-month-old male rat was used for Fig. 2E-H. One 2-month-old female rat, one 3-month-old female rat, and one 12-month-old male rat were used for Fig. 3A-D. A 9-month-old male rat was used for Fig. 4. Two 11-month-old male rats were used for Fig. 6. One 13-month-old female rat, one 5-month-old male rat, one 9-month-old male rat, one 12-month-old male rat, and one 13-month-old male rat were used for Fig. 7. Three male rats of unknown ages were used for Fig. 9. One 13-month-old female rat, one 8-month-old male rat, and one 13-month-old male rat were used for Fig. S4. Two 8-month-old male rats were used for Fig. S5A. Rats of unknown ages and sexes were used for Fig. S5B. All rats used for in vivo skin-penetration assays were outbred Sprague-Dawley rats (Rattus norvegicus Hsd:Sprague Dawley® SD®, Inotiv). Two 10-month-old female rats and seven 10-month-old male rats were used for skin-penetration assays in Fig. 3E-F.
Field-collected samples	N/A
Ethics oversight	All animal protocols and procedures were approved by the UCLA Office of Animal Research Oversight (Protocol ARC-2011-060). The protocol follows the guidelines set by the AAALAC and the Guide for the Care and Use of Laboratory Animals. Human skin samples were collected following approval by the University of California Institutional Review Board (Protocol 22-000400), with signed written informed consent obtained in accordance with the Declaration of Helsinki principles.

Note that full information on the approval of the study protocol must also be provided in the manuscript.

Plants

Seed stocks	N/A
Novel plant genotypes	N/A
Authentication	N/A
